# Introducing Our New Chief Editor

**DOI:** 10.1155/2020/4392067

**Published:** 2020-08-20

**Authors:** Mark Yorek

**Affiliations:** Department of Medicine, University of Iowa and Veterans Affairs Medical Center(VAMC), Room 204, Building 40, Iowa City, IA, USA

## Abstract

*Journal of Diabetes Research* is delighted to announce the installation of Dr. Mark Yorek as the new Chief Editor for the journal. In this Editorial, Dr. Yorek discusses his research background, his ideas for the journal's development, and his views on the direction of the field of diabetes.

It is with great pleasure that I begin my term as Chief Editor on the *Journal of Diabetes Research* by formally introducing myself and the aspirations I have for the journal. I am a Professor in the Department of Medicine at the University of Iowa and Associate Chief of Staff for Research at the Iowa City Veterans Affairs Medical Center. I received my PhD in Biochemistry in 1981 from the University of North Dakota in Grand Forks, North Dakota. This was followed by 3 years of postdoctoral training in the laboratory of Dr. Arthur Spector in the Department of Biochemistry at the University of Iowa, and in 1984, I joined the Department of Medicine. My training in graduate school was in carbohydrate metabolism, and my laboratory was the first to isolate and study rabbit hepatocytes at the time when everyone in the field was studying rat hepatocytes. My studies as a postdoctoral fellow at the University of Iowa focused on lipid metabolism, primarily omega-3 polyunsaturated fatty acids and the effect on membrane fluidity. As an independent investigator, I combined these interests and over the years have focused on the study of diabetes and complications primarily of the vasculature and peripheral nervous system. My laboratory has studied the effect of obesity and type 1 and type 2 diabetes on the vascular reactivity of epineurial arterioles of the sciatic nerve in relation to peripheral neuropathy. In relation to peripheral neuropathy, our tool box includes study of nerve conduction velocity, sensitivity of the cornea to a hyperosmotic solution, and nerve density in the skin and cornea. More recently, my interests have circled back to omega-3 polyunsaturated fatty acids and we are investigating the use of fish oil with or without salsalate as a potential treatment of diabetic peripheral neuropathy. We have also been examining the role of proresolving mediators of eicosapentanoic acid and docosahexaenoic acid on diabetic peripheral neuropathy. Soon, we hope to begin studies examining, first, the effect of fish oil supplementation with or without salsalate on the omega-3 index and the production of proresolving mediators in human diabetic subjects with or without neuropathy and matching control subjects. Following this work, we hope to begin clinical trials incorporating fish oil and salsalate as a safe and efficacious treatment for diabetic peripheral neuropathy.

I am excited to begin my role as Chief Editor of *Journal of Diabetes Research*. I look forward to working with the staff and the Editorial Board to further increase the visibility and ranking of the journal with the goal of making it one of the go-to journals for publication of original research articles and timely reviews in the field of diabetes, including appropriate articles in obesity and metabolic syndrome. As stated on the website, *Journal of Diabetes Research* is a peer-reviewed, Open Access journal that publishes research articles, review articles, and clinical studies related to type 1 and type 2 diabetes. The journal welcomes submissions focusing on the epidemiology, etiology, pathogenesis, management, and prevention of diabetes, as well as associated complications, such as diabetic retinopathy, neuropathy, and nephropathy. Without infringing on the focus areas of other journals that solicit articles relating to obesity and metabolic syndrome, another topic area that has been understudied and I think deserves a lot more attention is prediabetes. We know that complications can be detected in both animal models and human subjects with impaired glucose tolerance and insulin resistance. I think highlighting this topic area, along with those already stated, will serve the diabetes research community well. The approach of soliciting Guest Editors for the purpose of Special Issues is an excellent means to highlight areas of research that may be underrepresented or are in need of focused attention due to new and exciting advances in the field. This approach could be used to advance publication of research in many areas including prediabetes, insulin resistance, and postprandial glucose metabolism. The role of abnormal lipid metabolism is also an area of diabetes-related research that has not received enough attention. Elevated triglycerides and lipid dysregulation in general, and the effect it has in relation to type 2 diabetes and complications, is being recognized as an important problem in treating type 2 diabetic subjects. Abnormal lipid metabolism is likely a factor in prediabetes, and how dyslipidemia contributes to the complex etiology of type 2 diabetes deserves increased attention. Another topic area I would like to see promoted, although broad, would be clinical studies and advances in clinical research in diabetes. New technology and how this relates to advancing research in diabetes, both in animal models and human subjects, would also be an interesting Special Issue topic. The *Journal of Diabetes Research* is blessed by having a superb family of academic editors that excellently represent a diverse array of interests and expertise in the field of diabetes research. Working together, I think we can advance the important research that is being done by yourselves and our colleagues in diabetes.

Increasing exposure of the *Journal of Diabetes Research* is a key area of interest for me. I think we can all have a role in this goal. The impact factor for *Journal of Diabetes Research* saw a strong increase this year, and the journal holds a suitable acceptance rate of 31%. A common hurdle across Academic Publishing, and one that should be a focus for improvement in *Journal of Diabetes Research* is the time it takes for an author to get comments back from the initial review. There are several reasons that cause delays such as Academic Editors having difficulty assigning reviewers due to reviewer availability and increasing workloads. The journal does excellent work in providing a list of potential reviewers, and ensuring their contact information is regularly updated is key.

Another important priority for *Journal of Diabetes Research* is to support the Open Science mission, which is at the forefront of Hindawi's core goals, in order to remove barriers and allow researchers to make use of articles in new ways. Part of my role as Chief Editor, and for all those who work on the journal, will be to better support academic communities and continue to advocate Open Science as broadly as possible.

I look forward to working with the Academic Editors on bringing *Journal of Diabetes Research* to the next level in publishing the exciting work being done on this important worldwide problem.

*Mark Yorek*

